# Targeted Nutritional and Behavioral Feeding Intervention for a Child with Autism Spectrum Disorder

**DOI:** 10.1155/2016/1420549

**Published:** 2016-03-09

**Authors:** Kelly Barnhill, Amanda Tami, Claire Schutte, Laura Hewitson, Melissa L. Olive

**Affiliations:** ^1^The Johnson Center for Child Health and Development, Austin, TX 78701, USA; ^2^Applied Behavioral Strategies, New Haven, CT 06525, USA

## Abstract

A variety of feeding issues and concerns, including food aversion, food selectivity, and complete food refusal, are not uncommon among children with autism spectrum disorder (ASD). Other underlying issues are often comorbid with the concerns for feeding and ASD. These may include food allergies, gastrointestinal issues, oral motor issues, and swallowing disorders. The refusal to consume particular foods coupled with the inability to tolerate, digest, and absorb these foods can compromise an individual's overall nutrition status. Therefore, a child's behavior toward food and feeding activities has great impact on dietary intake, nutritional status, and growth. This case report is the first to document combined medical, behavioral, and nutritional intervention for a toddler with ASD and comorbid feeding disorder.

## 1. Introduction

In 1943, Kanner first described feeding difficulties among children with autism spectrum disorder (ASD) [1]. Of the 11 descriptive cases in his paper, six children experienced severe feeding difficulties. Since then, others have reported similar patterns among children with ASD [2–4].

While the current medical model allows for billing of feeding therapy for children with ASD by occupational therapists or speech and language therapists, peer-reviewed publications on the success of these strategies are sparse. Meanwhile, most of the peer-reviewed scientific studies on feeding therapy for children with ASD consist of behavioral intervention. Specifically, in their review, Matson et al. [3] noted that a variety of behavioral interventions have been attempted including behavioral momentum, response cost, simultaneous presentation, sequential presentation, and differential reinforcement of alternative behavior (DRA).

Additionally, in 1999, Kerwin reviewed the literature on treatments for pediatric feeding problems across all populations including ASD [5]. She reported that, out of 79 studies, the majority of studies employed behavioral intervention procedures. She, too, listed a variety of behavioral procedures including differential attention (i.e., attending to children when they are eating and ignoring inappropriate meal time behavior), positive reinforcement for eating, and planned consequences for inappropriate meal time behavior (e.g., extinction, nonremoval of the spoon). Thus, a behavioral approach to feeding interventions has been well established for children with ASD as well as for children without ASD [6].

To date, few studies have reported the use of nutritional intervention as part of, or as the primary component of, a feeding intervention. Thus, little is known regarding the effectiveness of nutritional intervention to address feeding difficulties. Therefore, the purpose of this paper is to describe the results of a clinical case study regarding the outcomes of an intensive, short-term nutritional and behavioral outpatient feeding program for a toddler with ASD.

## 2. Case Presentation

### 2.1. Participant and Developmental History

The participant, a 28-month-old female previously diagnosed with ASD, presented for a short-term outpatient nutritional and behavioral feeding intervention program.

The participant's mother reported a full term pregnancy, delivered with shoulder dysplasia, but otherwise no further complications. She scored an 8/9 on the Appearance, Pulse, Grimace, Activity, and Respiration (APGAR) test. The mother experienced significant hyperemesis gravis throughout the pregnancy and was maintained on Zofran from 6 to 40 weeks. Additionally, she developed a life-threatening allergy to legumes, nuts, pole beans, and peas during her pregnancy. At the time of intake, the mother reported no other complications or concerns involving pregnancy and childbirth.

Pediatric records and parental reports stated that the participant met all physical developmental milestones in appropriate windows of time. She rolled over, crawled, and walked as expected. By 10 months of age, the mother noted that the participant cooed, babbled, and used sounds as word approximations, and her vocabulary included 5-6 simple words. The participant's language development continued to progress typically until just past 12 months of age, when she lost all language (babbling, sounds, word approximations, and words). The participant's mother reported that she became less engaged. She also reported that the participant had an upper respiratory virus during the same period of time, which also included a fever of over 101 degrees.

The participant's pediatrician completed an M-CHAT screening and referred her for a developmental evaluation at 15 months of age. At 17 months of age, the participant received an initial evaluation and met criteria for ASD and Sensory Processing Disorder (SPD). The participant subsequently began receiving speech and language intervention for 30 minutes twice weekly.

In the area of eating, the participant's mother noted her to be a curious eater prior to the loss of language and onset of ASD symptoms. She stated that her daughter tried many new foods, had several favorites, and requested food using gestures and autistic leading even after losing her language. Pediatric records revealed that the participant ate solid baby food by 8 months of age, and she transitioned to mashed-up table foods served at the family meal by 10 months of age. However, by the time of the initial referral evaluation at 15 months of age, the participant developed food selectivity and she began refusing food altogether.

Thus, she received a referral for occupational therapy (OT). Records reveal that the participant engaged in multiple tantrums and meltdowns over the course of the OT evaluation. At age of 23 months, she showed severe fine motor delays in the areas of grasping (age equivalence [AE] of 4 months) and visual-motor integration (AE = 11 months) skills. The evaluator also noted that the participant demonstrated decreased bilateral strength and poor endurance with all tasks.

At 20 months of age, she began receiving OT to address feeding concerns. The participant received therapy for 30 minutes one time per week for over 6 months with no notable progress. Records indicate that therapy consisted of the Get-Permission Trust Approach [7] and food chaining [8]. It should be noted that neither of these approaches has been studied empirically. Therapy records indicate that the participant made great progress in speech and language with these therapies but she experienced no changes in her feeding behavior.

At 26 months of age, the participant became ill with influenza resulting in complete food refusal and hospitalization for 48 hours for rehydration support. The participant's mother also became ill with influenza in this time frame. Shortly after, her mother contacted our center for assistance.

### 2.2. Setting and Materials

The staff conducted most therapy sessions in a small office with one door but no windows. During therapy, the participant sat in a child-size chair in front of a child-size table flanked by her mother and the therapist. The therapist kept one plate of food off to the side and out of the child's reach and a second plate and appropriate utensil sat in front of the child. The staff placed extra cups, plates, utensils, and paper towels on an adjacent chair or table.

Following skill acquisition, the staff moved therapy sessions to the family's hotel suite and then finally to a casual restaurant. The nutritional staff instructed the participant's mother regarding what types of food to bring to therapy as well as what food to order in the restaurant setting.

### 2.3. Measurement

The behavioral staff collected a variety of data each session. Operational definitions for each measure are listed in Table 1. Therapists recorded continuous measures for each behavior each time the therapist or parent presented a bite to the participant.

### 2.4. Procedures and Experimental Design

The staff collected data throughout intervention sessions to measure changes in eating behaviors. We opted for a clinical case study rather than an experimental design for several reasons. First, the services described in this evaluation were of a truly clinical nature. Second, speed was a high priority due to medical urgency to prevent dehydration again. And finally, limited family finances associated with staying in a hotel, traveling from another location in order to receive therapy, and preparing foods for therapy each day prevented the research team from taking the additional time necessary to demonstrate experimental control. Based on these factors, a true single subject experimental design was not deemed appropriate.

### 2.5. Intake

The staff completed an intake with the participant's mother lasting 60 to 90 minutes about two weeks prior to beginning therapy. The staff used the intake to obtain detailed information regarding the participant's feeding, developmental, and behavioral history. As part of the intake, the mother also completed a 3-day food diary. Per the 3-day food diary the participant's daily caloric intake aside from breast milk included approximately 125 calories from fewer than 10 foods such as a banana, chicken nugget, or a fruit-and-vegetable-based smoothie. In addition to table foods, the participant's mother reported an average of 80 minutes of breastfeeding across the course of a day.

Unfortunately, the staff could not quantify and qualify the nutritional content of the mother's breast milk. Under circumstances such as these, it is important to accurately assess the mother's dietary intake to better understand the nature and quality of her breast milk. However, previous studies have shown that the nutritional qualities of breast milk vary by a number of factors, including stage of lactation, term or preterm infancy, and individual dietary intake [9].

The participant's mother reported that she refused to come near unknown foods and screamed, cried, and had tantrums in the presence of foods when others were eating. The participant's mother also noted that if food entered her mouth, the participant made herself gag until the point of emesis. The participant's mother also reported that she utilized a spoon to feed herself and used a fork to get food to her mouth. However, she noted the child required assistance in getting food on the fork. At the time of intake, the participant refused all liquids other than breast milk, with the exception of tiny sips of water on very rare occasions. Her mother reported that the participant could drink breast milk from a cup with no assistance. She noted that her daughter could drink from a straw, but she often choked.

Additional behavioral concerns reported at the time of clinical intake included sleep disturbance, toe-walking, spinning, hitting, hair pulling, problems with transitions, and excessive meltdowns. Medical concerns included constipation and eczema.

### 2.6. Preference Assessment

Before beginning the intensive feeding therapy, the staff observed the participant as she played in a large playroom with a variety of toys to determine potential reinforcers. The staff selected several toys and activity items with which the participant spontaneously played. Items included cotton balls (pom-poms), bubbles, a dry erase board and marker, and iPad with access to YouTube videos. The staff varied reinforcers during each session based on the child's preferences.

### 2.7. Diagnostic Evaluation

A licensed psychologist completed a diagnostic evaluation using the Autism Diagnostic Observation Schedule-Second Edition (ADOS-2), Toddler Module. The ADOS-2 is a directly administered, standardized measure used to evaluate the presence or absence of behaviors associated with ASD. The Toddler Module results in cutoff scores indicating a “range of concern” that provides an estimate of the level of concern for the presence of ASD. This includes little-to-no concern, mild-to-moderate concern, and moderate-to-severe concern. The participant met the mild-to-moderate range of concern suggestive of symptoms of ASD.

The psychologist also completed the Autism Diagnostic Interview-Revised (ADI-R) with the participant's mother. The child's scores on the ADI-R met the classification for autism. Therefore, based on the record review, current testing, and the criteria outlined in the Diagnostic and Statistical Manual of Mental Disorders, Fifth Edition (DSM-5), the psychologist assigned a diagnosis of ASD.

### 2.8. Medical Evaluation

A physician on the team evaluated the participant at the onset of feeding therapy. On physical examination, he assessed her vitals along with a weight of 25 lbs. and height of 33.7 inches. This placed the participant in the 10th-to-15th percentile for both weight and height. Thus, the participant did not meet criteria for failure to thrive. Her anthropometric measurements fell at the 25th percentile. Bloodwork completed at the time of feeding clinic initiation indicated that the participant had iron deficiency anemia and low serum vitamin D (14 ng/mL).

### 2.9. Intervention

After the feeding intake, the staff informed the participant's mother of intervention procedures, specifically not to feed her two hours before or after each therapy session. This is known as appetite inducement [10]. The participant subsequently started feeding therapy and engaged in three sessions per day for 4 days. Each session lasted for approximately one hour after she sat in her chair or until she consumed between 40 and 60 bites, whichever came first. The staff scheduled sessions at approximate meal times (e.g., breakfast at 8 a.m., lunch at 11, and dinner at 3 p.m.). The staff discharged the participant when she met the behavioral criteria of accepting 80% or more bites within five seconds of presentation with zero instances of challenging behavior.

The staff initially put single bites on the empty plate and instructed the participant to eat. As independent eating increased, staff put multiple bites of each food on the plate so that foods would mix and touch. The participant did not have access to all of the food for her meal at once in case protesting or other disruptive behavior resulted in the plate being tipped or the food otherwise spilled. She also had access to an open cup of water with a straw.

For all intervention procedures, the staff allowed the participant to sample preferred reinforcers for about one minute prior to intervention. Following exposure to the reinforcer, the staff counted down from 5 to 1 and told her, “Ok, now we are going to put this away. You may have it again, after you eat.” For initial intervention sessions, the staff allowed the participant access to reinforcement for 30 seconds following each successful bite. The staff faded this reinforcement over the course of the week so that she would eat an entire meal prior to receiving access to reinforcement.

The intervention package consisted of shaping, differential reinforcement, prompting, and escape extinction. The staff used a shaping technique to help the participant access reinforcement while improving her acceptance of novel food. The staff modeled each step prior to asking the child to comply. Specifically, the staff asked the participant to touch the food to her lips. This was followed by touching the food to the tongue and finally placing the food in the mouth to chew and swallow. When she engaged in the target behavior on 80% of bite presentations with zero instances of challenging behavior, the staff targeted the next step in the shaping sequence until she chewed and swallowed each bite.

The staff also utilized a procedure known as escape extinction. Specifically, the staff did not remove the bite until the child engaged in the appropriate behavior targeted from the shaping sequence. If she did not engage in the behavior independently, the staff used light physical prompting to pick up the utensil. If she refused physical prompting then the therapist held the utensil next to her lips and waited for her to willingly accept the bite. This has been referred to in the literature as nonremoval of the spoon/fork [5, 11]. Finally, the staff utilized differential reinforcement of alternative behavior (DRA) [5]. In this technique, the staff presented the participant with a single bite of food and instructed her to “take a bite (or the target behavior in the shaping sequence) and then you can have more_.” Once she engaged in the target behavior, she received verbal praise and 30 seconds' access to the reinforcer. The staff taught the participant's mother to cheer and provide verbal praise to her when she tried new foods.

### 2.10. Parent Training

Parent training consisted of modeling feeding therapy followed by live coaching and feedback, followed by generalization of therapy to a natural environment and to other caregivers. Specifically, the staff required parent presence during each feeding session. During initial feeding sessions, we asked her mother to “sit quietly” and “cheer loudly.” Specifically, we instructed her to watch the session and to avoid interacting with the participant until she accepted her bite (or the target behavior in the shaping sequence).

When she engaged in appropriate chewing and swallowing behavior for 80% of bite presentations with zero (or near-zero) challenging behavior, we asked the parent to take over therapy and the therapist continued to collect all data. The therapist reminded the parent to follow the procedures. Specifically, the staff reminded parents to tell the child to “eat *X* number of bites and then you can have_.” The staff reminded parents to limit verbal coaxing to take bites.

## 3. Results

Table 2 displays the list of foods the participant consumed over the course of treatment. For the first session, she protested, cried, and eloped from the table for 49 minutes. She remained at the table for the rest of the session. The second feeding session followed the same process. She sat in her chair and accepted her first bite within 15 minutes of the session start time. As can be seen, the child quickly improved to sitting for the entire meal.

**Figure 1:**
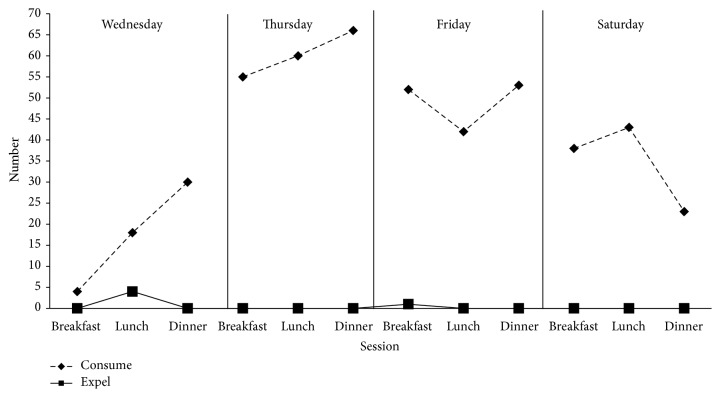
Total bites consumed and expelled.

Figure 1 shows the number of bites consumed and expelled for each meal. On the first day, she consumed 18 bites out of the 22 presented. She expelled the other 4 bites, 3 of which were due to gagging resulting in emesis. For each remaining meal, the staff allowed her to lick only new foods that she had not tried before. The participant's mother took over intervention procedures on lunch of day 2. The staff transitioned therapy to the hotel room on day 3. Also on day 3, her father and grandmother learned to implement procedures and she demonstrated an ability to accept new foods with both of them. The staff generalized feeding therapy to a restaurant on day 4. The participant met the behavioral criterion in 4 days.

**Figure 2:**
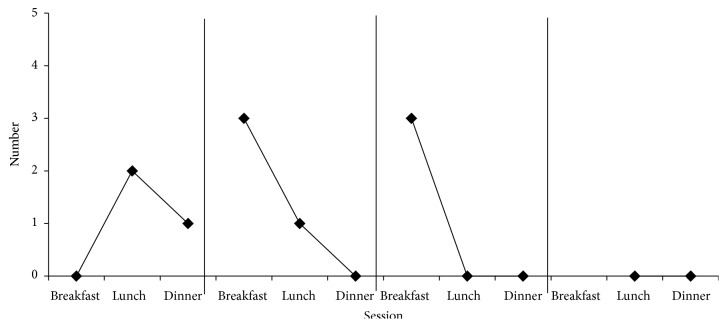
Frequency of gags and emesis.

Figure 2 shows the number of times that the participant gagged and had emesis during each session. Gagging peaked at 3 times during the fourth session (breakfast on day 2) and then stopped. She gagged again three times during breakfast on day 3; however, at this time, gagging followed by emesis occurred at the end of the meal and in response to foods that the participant had eaten previously with no gagging.

**Figure 3:**
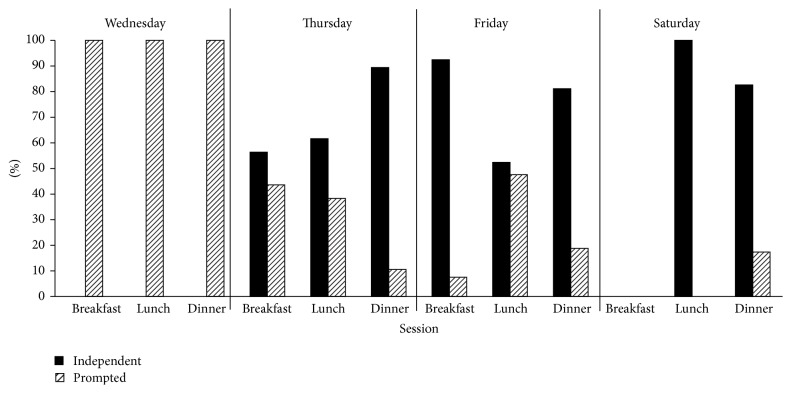
Percent of bites taken independently.

Figure 3 shows the percentage of bites that the participant accepted within 5 seconds. The data shows an increase in independent bites from 0% to a rate consistently above 80% beginning with the sixth session (dinner on day 2). The exception to this rate is lunch on day 3, which was the first session for which the participant's father implemented intervention.

## 4. Discussion

We report the results of a case study of a toddler with ASD, a feeding disorder, and complicated nutritional issues. The results of this case study support the effectiveness of a multidisciplinary intervention consisting of behavioral intervention, nutritional intervention, and medical support. The participant in this study rapidly increased her food acceptance and consumption. She went on to generalize the skills to her parents and her grandmother and to other settings. While other studies have reported maintenance and generalization, a need exists to carefully document and report such changes [5]. Moreover, to our knowledge, no other study has documented the effects of a combined behavioral and nutritional intervention. Additionally, we were unable to locate any studies documenting the effects of feeding therapy as a component of difficulties weaning from breast milk.

Not only did we see increases in appropriate feeding behaviors such as self-feeding, sitting appropriately for the meal, and eating new foods, but also we saw decreases in challenging behavior as a result of intervention. Crying, screaming, throwing the plate/food, and tantrums all decreased to zero rates. The results from this study add to the existing literature on the effectiveness of behavioral treatment for food selectivity [6, 12].

When feeding intervention is warranted for children under the age of two, professionals should use caution and ensure that the toddler has been properly weaned from nursing and that appropriate table food intake has been achieved. Given that it is nearly impossible to measure nutritional intake from breastfeeding, additional research in this area is clearly needed.

Finally, we must discuss the role of a multidisciplinary approach. Before attempting any feeding intervention, children should be screened by a variety of professionals to better understand the underlying causes of the feeding problem. Berry and colleagues suggest a multidisciplinary team in recently published guidelines [13]. Within the medical field, the need for a gastrointestinal evaluation should be assessed. Things to consider include a history of reflux, diarrhea, constipation, and presence of blood in the stool. Professionals should ascertain the need for a workup to determine food allergies and sensitivities. Considerations should include skin conditions such as rashes and eczema, side effects such as itching and throat irritation. An oral motor evaluation should be completed to assess oral motor and swallowing functions. A nutritional workup should be completed to determine nutritional deficiencies. Finally, a behavioral assessment should be included to assist in determining if the feeding concerns have a behavioral component.

Results of this case study should be viewed with caution due to case study limitations. First, this was not a research study and we did not employ an experimental design. However, it is unlikely that any other external event could have influenced the child's change in eating behavior. Another limitation is that the participant and her parents were not from a random sample; they sought out behavioral treatment from our center. Thus, the results in this case study could be partially attributed to parent resources, personality type, or similar explanation.

The outcomes of this evaluation suggest that short-term intensive outpatient feeding therapy for children with food selectivity can be highly successful. Given the rapidity with which outcomes were obtained, a short-term intensive model may result in decreased costs and decreased stress for families. Moreover, given the potential benefits of this model and the lack of available feeding services in general, additional research is needed on the creation of systems for disseminating and expanding the availability of multidisciplinary therapy programs such as the one described here.

The participant's mother also presented with a high amount of stress. The staff also reported difficult interactions with her mother and noted that stress appeared to be involved. For example, one morning, the mother noted her fear that the child was becoming dehydrated again (this was the morning following an episode of emesis at dinner the previous evening). Despite consultation with the physician who noted that the participant did not present symptoms of dehydration, the mother nursed her child prior to feeding intervention that morning, even though this did not comply with the feeding protocol. Clearly, the intervention caused stress for her mother. While stress has been evaluated in other studies [14, 15], the role of stress before, during, and after intensive feeding therapy as well as the effects of counseling support during and after therapy should be evaluated.

In conclusion, this study is the first of its kind highlighting the importance of a multidisciplinary approach to address pediatric feeding problems. Parents should be informed of the effectiveness of the behavioral approach and behavioral providers should be highlighted as a source of appropriate therapy for these types of concerns. Additionally, nutritional, medical, and oral motor assessments are critical components of a feeding program, and the skills and expertise of therapists should be utilized in the development and implementation of pediatric feeding programs. Finally, comprehensive multidisciplinary programs should consider the inclusion of counseling services in order to address the stressors related to intensive feeding therapy.

## Figures and Tables

**Table 1:** Operational definitions of child behaviors.

Behavior	Definition
Bite acceptance	Opening mouth 1.3 cm or wider within 5 s of the bite presentation and allowing food placement in mouth Placing food in mouth within 5 s of bite presentation
Bite consumption	Swallows bite and mouth is clean of food
Expulsion	Appearance of food past the outer edge of lips following an acceptance
Gag	Open mouth accompanied with guttural sound; regurgitative spasm in the throat
Vomit	To eject part or all of stomach contents through the mouth
Aggression	Any physical contact from child to therapist, supervisor, or parent
Disruption	Includes physical refusal, verbal refusal, and vocal refusal
Physical refusal	Turning head when bite is presented or pushing away plate, fork, spoon, or cup
Verbal refusal	Child verbally states that he does not want the food
Vocal refusal	Screaming or making vocal sound contingent on bite presentation

**Table 2:** New foods log.

Meal	Day	Food
Breakfast	1	Banana, oatmeal with raisins, chicken sausage, juice, water
Lunch	1	Strawberries, carrots, chicken, French fries, sun butter, juice
Dinner	1	Salmon, rice, broccoli, peaches

Breakfast	2	Egg, ham, oranges, juice
Lunch	2	Apple, turkey, avocado, fritter, tomato, juice
Dinner	2	Zucchini, raspberry, noodle, meat, noddle/meat mixture

Breakfast	3	Sausage, egg, toast, avocado, egg/sausage mixture, juice
Lunch	3	Hamburger, beans with chips, cantaloupe, spinach
Dinner	3	Blackberry, cauliflower, bean and cheese quesadilla, juice

Breakfast	4	(Parents fed without therapist assistance)
Lunch	4	Roast beef, raspberry, spinach, lentil chips
Dinner	4	Taco bowl (black bean, brown rice, tomato, guacamole, cheese, sour cream mix)
